# Microbes as Engines of Ecosystem Function: When Does Community Structure Enhance Predictions of Ecosystem Processes?

**DOI:** 10.3389/fmicb.2016.00214

**Published:** 2016-02-24

**Authors:** Emily B. Graham, Joseph E. Knelman, Andreas Schindlbacher, Steven Siciliano, Marc Breulmann, Anthony Yannarell, J. M. Beman, Guy Abell, Laurent Philippot, James Prosser, Arnaud Foulquier, Jorge C. Yuste, Helen C. Glanville, Davey L. Jones, Roey Angel, Janne Salminen, Ryan J. Newton, Helmut Bürgmann, Lachlan J. Ingram, Ute Hamer, Henri M. P. Siljanen, Krista Peltoniemi, Karin Potthast, Lluís Bañeras, Martin Hartmann, Samiran Banerjee, Ri-Qing Yu, Geraldine Nogaro, Andreas Richter, Marianne Koranda, Sarah C. Castle, Marta Goberna, Bongkeun Song, Amitava Chatterjee, Olga C. Nunes, Ana R. Lopes, Yiping Cao, Aurore Kaisermann, Sara Hallin, Michael S. Strickland, Jordi Garcia-Pausas, Josep Barba, Hojeong Kang, Kazuo Isobe, Sokratis Papaspyrou, Roberta Pastorelli, Alessandra Lagomarsino, Eva S. Lindström, Nathan Basiliko, Diana R. Nemergut

**Affiliations:** ^1^Institute of Arctic and Alpine Research, University of Colorado Boulder, BoulderCO, USA; ^2^Biological Sciences Division, Pacific Northwest National Laboratory, RichlandWA, USA; ^3^US Department of Energy, Joint Genome Institute, Walnut CreekCA, USA; ^4^Department of Forest Ecology, Federal Research and Training Centre for Forests, Bundesforschungs- und Ausbildungszentrum für WaldVienna, Austria; ^5^Department of Soil Science, University of Saskatchewan, SaskatoonSK, Canada; ^6^Helmholtz Centre for Environmental Research – Centre for Environmental BiotechnologyLeipzig, Germany; ^7^Department of Natural Resources and Environmental Sciences, University of Illinois at Urbana-Champaign, UrbanaIL, USA; ^8^Life and Environmental Sciences and Sierra Nevada Research Institute, University of California – Merced, MercedCA, USA; ^9^School of Medicine, Flinders University, AdelaideSA, Australia; ^10^Institut National de la Recherche Agronomique – AgroecologyDijon, France; ^11^Institute of Biological and Environmental Sciences, University of AberdeenAberdeen, UK; ^12^Irstea, UR MALY, Centre de Lyon-VilleurbanneVilleurbanne, France; ^13^Department of Biogeography and Global Change, Museo Nacional de Ciencias Naturales, Consejo Superior de Investigaciones CientíficasMadrid, Spain; ^14^Environment Centre Wales, Bangor UniversityGwynedd, UK; ^15^Department of Microbiology and Ecosystem Science, University of ViennaVienna, Austria; ^16^Häme University of Applied SciencesHämeenlinna, Finland; ^17^School of Freshwater Sciences, University of Wisconsin-Milwaukee, MilwaukeeWI, USA; ^18^Department of Surface Waters, Eawag: Swiss Federal Institute of Aquatic Science and TechnologyKastanienbaum, Switzerland; ^19^Centre for Carbon, Water and Food, The University of Sydney, SydneyNSW, Australia; ^20^Institute of Landscape Ecology, University of MünsterMünster, Germany; ^21^Department of Environmental and Biological Sciences, University of Eastern FinlandKuopio, Finland; ^22^Natural Resources InstituteVantaa, Finland; ^23^Institute of Soil Science and Site Ecology, Technische UniversityDresden, Germany; ^24^Institute of Aquatic Ecology, Facultat de Ciències, University of GironaGirona, Spain; ^25^Institute for Sustainability Sciences – AgroscopeZurich, Switzerland; ^26^CSIRO Agriculture Flagship, CraceACT, Australia; ^27^Department of Biology, University of Texas at Tyler, TylerTX, USA; ^28^EDF R&D, National Hydraulics and Environmental LaboratoryChatou, France; ^29^Department of Microbiology and Ecosystem Science, University of ViennaVienna, Austria; ^30^Division of Terrestrial Ecosystem Research, Department of Microbiology and Ecosystem Science, University of ViennaVienna, Austria; ^31^Department of Ecosystem and Conservation Sciences, University of Montana, MissoulaMT, USA; ^32^Centro de Investigación y Docencia Económicas – Consejo Superior de Investigaciones CientíficasValencia, Spain; ^33^Department of Biological Science, Virginia Institute of Marine Science, Gloucester PointVA, USA; ^34^AES School of Natural Resources Sciences, North Dakota State University, FargoND, USA; ^35^LEPABE - Laboratory for Process Engineering, Environmental, Biotechnology and Energy, Faculdade de Engenharia da Universidade do PortoPorto, Portugal; ^36^Southern California Coastal Water Research Project Authority, Costa MesaCA, USA; ^37^UMR, Interactions Sol Plante Atmosphère, INRA BordeauxVillenave d’Ornon, France; ^38^Department of Forest Mycology and Plant Pathology, Swedish University of Agricultural SciencesUppsala, Sweden; ^39^Department of Biological Sciences, Virginia Polytechnic Institute, State University, BlacksburgVA, USA; ^40^Centre Tecnològic Forestal de CatalunyaSolsona, Spain; ^41^Centre de Recerca Ecològica i Aplicacions Forestals, Cerdanyola del VallèsBarcelona, Spain; ^42^School of Civil and Environmental Engineering, Yonsei UniversitySeoul, South Korea; ^43^Department of Applied Biological Chemistry, The University of TokyoTokyo, Japan; ^44^Department of Biomedicine, Biotechnology and Public Health, University of CadizPuerto Real, Spain; ^45^Research Centre for Agrobiology and PedologyFlorence, Italy; ^46^Department of Ecology and Genetics/Limnology, Uppsala UniversityUppsala, Sweden; ^47^Vale Living with Lakes Centre and Department of Biology, Laurentian University, SudburyON, Canada; ^48^Biology Department, Duke University, DurhamNC, USA

**Keywords:** microbial diversity, functional gene, statistical modeling, microbial ecology, ecosystem processes, respiration, nitrification, denitrification

## Abstract

Microorganisms are vital in mediating the earth’s biogeochemical cycles; yet, despite our rapidly increasing ability to explore complex environmental microbial communities, the relationship between microbial community structure and ecosystem processes remains poorly understood. Here, we address a fundamental and unanswered question in microbial ecology: ‘When do we need to understand microbial community structure to accurately predict function?’ We present a statistical analysis investigating the value of environmental data and microbial community structure independently and in combination for explaining rates of carbon and nitrogen cycling processes within 82 global datasets. Environmental variables were the strongest predictors of process rates but left 44% of variation unexplained on average, suggesting the potential for microbial data to increase model accuracy. Although only 29% of our datasets were significantly improved by adding information on microbial community structure, we observed improvement in models of processes mediated by narrow phylogenetic guilds via functional gene data, and conversely, improvement in models of facultative microbial processes via community diversity metrics. Our results also suggest that microbial diversity can strengthen predictions of respiration rates beyond microbial biomass parameters, as 53% of models were improved by incorporating both sets of predictors compared to 35% by microbial biomass alone. Our analysis represents the first comprehensive analysis of research examining links between microbial community structure and ecosystem function. Taken together, our results indicate that a greater understanding of microbial communities informed by ecological principles may enhance our ability to predict ecosystem process rates relative to assessments based on environmental variables and microbial physiology.

## Introduction

The links between complex environmental microbial communities and ecosystem processes remain unclear (Carney and Matson, 2005; Prosser et al., 2007; van der Heijden et al., 2008; Petersen et al., 2012; Wallenstein and Hall, 2012; Graham et al., 2014; Martiny et al., 2015), and an emerging field of research has begun to investigate the utility of microbial data for improving predictions of carbon (C) and nitrogen (N) cycling beyond estimates based solely on environmental data (Todd-Brown et al., 2012; Wieder et al., 2013; Reed et al., 2014; Powell et al., 2015). While researchers have attempted to enhance ecosystem process models by parameterizing microbial physiological properties such as drought tolerance (Manzoni et al., 2014), growth efficiency (Hagerty et al., 2014), dormancy (Wang et al., 2015), and turnover rates (Wieder et al., 2013), these models often fail to consider variation in microbial community structure that may regulate ecosystem process rates (Bouskill et al., 2012; Kaiser et al., 2014). As such, we still lack an integrated understanding of the interactions between microbial communities and ecosystem function, and a central question in ecosystem science remains: ‘Under what circumstances does information on microbial communities add to our predictive power of ecosystem processes?’ Addressing this question is essential not only for improving knowledge on how critical biogeochemical cycles may respond to current and impending environmental change, but also for allowing us to identify factors that determine microbial community structure and activity in space and time.

The specific conditions in which microbial community structure – broadly defined here as information on diversity and/or abundance of taxa within a community – may improve predictions of ecosystem process rates beyond models based on environmental and physiological attributes varies with a myriad of biotic and abiotic factors (Knelman and Nemergut, 2014; Nemergut et al., 2014). Research has demonstrated global patterns in microbial communities that correlate with environmental factors such as salinity (Lozupone and Knight, 2007; Auguet et al., 2010), pH (Lauber et al., 2009), and habitat type (Dinsdale et al., 2008; Nemergut et al., 2011; Fierer et al., 2012), and links between microbial community and ecosystem processes have been observed within numerous individual studies (van der Heijden et al., 1998; Torsvik and Øvreås, 2002; Bardgett and van der Putten, 2014; Wagg et al., 2014). However, these relationships and the underlying ecological principles that generate them may vary among environments, relevant microbial traits, and processes of interest (Wallenstein and Hall, 2012; Nemergut et al., 2014). For example, stochastic assembly processes (Hubbell, 2001; Rosindell et al., 2011; Stegen et al., 2012; Nemergut et al., 2013), phenotypic plasticity (DeWitt et al., 1998), and priority effects (Fukami, 2004; Fukami et al., 2010) can decouple microbial community structure from environmental conditions, and under such conditions, microbial community structure should be central to explaining ecosystem process rates. A unifying perspective that accounts for the dynamic relationships between microbial community structure and ecosystem function is vital for improving predictions of ecosystem process rates, yet recent work has been largely unable to decipher coherent trends in such relationships across studies (Rocca et al., 2014; Bier et al., 2015).

Thus, to assess the importance of microorganisms in explaining ecosystem processes, we used statistical modeling to evaluate the value of environmental variables and microbial community structure both alone and in conjunction for explaining rates of C and N cycling processes within global datasets. Although the literature reporting environmental variables, microbial community data, and biogeochemical processes in a single study is sparse (Rocca et al., 2014), we collected and re-analyzed 82 datasets spanning a multitude of diverse environments. We hypothesized that while the environment is a strong driver of most ecosystem processes, microbial community structure adds to our understanding of processes under certain circumstances, and we discuss patterns across ecological, biogeochemical, and phylogenetic subsets of data. Our analysis generates new insights about the relationship between microbial community structure and a variety of ecosystem processes.

## Materials and Methods

We completed an exhaustive literature review for studies measuring environmental variables, microbial community structure, and biogeochemical processes across a range of ecosystems (Supplementary Table S1). We then constructed statistical models for three predictor sets and their additive combinations – environmental data, microbial community data, and microbial biomass measurements (Supplementary Figure S1). Because the measurement of microbial biomass (defined as microbial C and/or N content) was not a criterion for dataset selection, only 28 datasets included biomass measurements. To incorporate differences in microbial community structure into our models, we used ordination-based techniques to condense multivariate community data into vectors that we included as predictors in our models (Supplementary Methods). The Shannon diversity index of each sample was also included as a measure of alpha diversity. In total, these taxonomic metrics are referred to as ‘community diversity’ when applied to 16S rRNA genes or PLFAs and ‘functional diversity’ when applied to functional genes. Functional gene abundances were included as measures of total gene abundance per gram of soil when possible (*n* = 17), although five datasets only listed gene abundances as normalized relative to 16S rRNA gene abundance.

Within each type of data, methodology to collect environmental, microbial, and process data, as well as variables collected, inevitably varied. To minimize potential error introduced by these differences, we re-analyzed data within each dataset using a multimodel inference approach with the ‘MuMIn’ package (Barton, 2011) in *R* software (R Core Team, 2014) and subsequently compared results from our analyses. Multimodel inference is a broad regression-based, model-averaging statistical approach designed to reduce errors in model selection, and this method has the advantage of standardizing our approach across studies while accounting for a lack of *a priori* system-specific knowledge (Burnham and Anderson, 2002). This statistical approach has been used in other small scale studies with similar objectives (Powell et al., 2015). We validated the accuracy of MuMIn in our dataset by comparing models for several datasets with expert-built regression models, which yielded comparable results (more details available in Supplementary Methods).

Using the ‘dredge’ command in the ‘MuMIn’ package to fit and evaluate the explanatory power of all possible combinations of variables within a predictor set on a process rate, we selected a set of best fit models for each predictor set consisting of all models with a delta AICc value no more than four greater than the model with the lowest AICc value (Burnham and Anderson, 2004). We generated an averaged final model from this model set using Akaike’s weights, implemented with the ‘model.avg’ command in *R* (Supplementary Methods). Final models from different predictor sets were compared for statistically significant differences at a delta AICc value of four to provide conservative estimates of model improvements (Burnham and Anderson, 2004). Models were also evaluated for ecologically relevant improvement, defined by an increase in adjusted *R^2^* value greater than 10% of the environmental model adjusted *R^2^* value. This criterion was implemented to remove artifacts from datasets in which statistically different models, based on AICc values, yielded similar adjusted *R^2^* values. Only models that showed both statistical and ecological improvement were considered to be improved.

Finally, model results were synthesized across studies within ecologically relevant subsets of data. We examined results within the full dataset and within biogeochemical process [C mineralization (referred to here as ‘respiration’), nitrification, denitrification, N mineralization], microbial data (PLFA, tRFLP, ARISA, DGGE, qPCR, next generation sequencing) and environment types (natural soil, sediment, agricultural soil) with sufficient replication (*n* ≥ 12). We report the mean adjusted *R^2^* of models and the increase in adjusted *R^2^* value as measures of effect size as well as the percent of models statistically improved by the addition of microbial data relative to models constructed with only environmental parameters. Differences among the explanatory power of models with environmental, microbial, or both environmental and microbial data were assessed by comparing the distribution of model adjusted *R^2^* values within each predictor set using unpaired one-sided Mann–Whitney *U*-tests for non-parametric data. We also examined correlations between predictor sets to determine the extent to which environmental variables explained variation in microbial community structure and biomass and to which biomass explained variation in microbial community structure. We analyzed these relationships with redundancy analysis (RDA), utilizing the ‘ordistep’ function in the ‘vegan’ package (Oksanen et al., 2013) in *R* to automate forward model selection, and we report the average *R^2^* values of correlations between predictor sets as a measure of effect size (Supplementary Methods).

## Results

**FIGURE 1:**
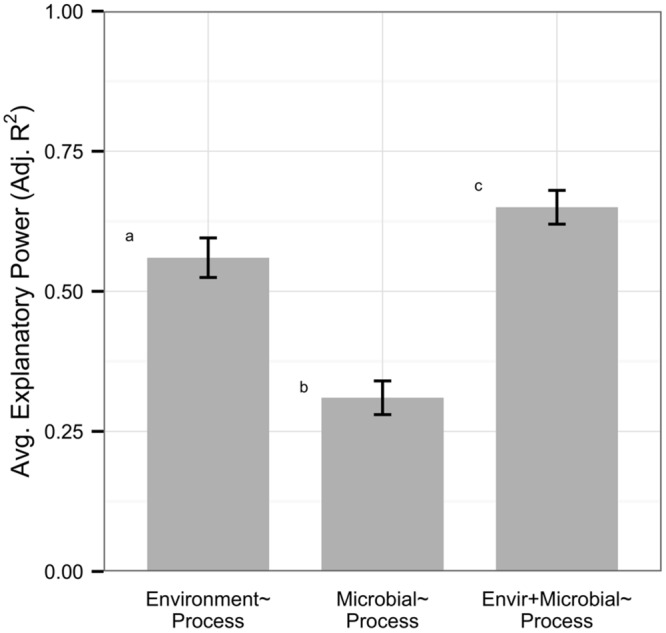
**Within the full dataset (*n* = 82), environmental variables explained more variation in ecosystem process rates than microbial community structure.** Microbial predictors alone had low explanatory power on processes but improved model explanatory power on average. Error bars denote standard errors, and letters represent significantly different groups (Mann–Whitney *U, p* < 0.05).

Overall, models based on environmental variables explained significantly more variation in processes than models based on microbial community structure (*n* = 82, average adjusted *R^2^* 0.56 vs. 0.31, Mann–Whitney *U, p* < 0.0001, **Figure 1**). On average, models with both environmental and microbial predictors explained more variation in processes than environmental models (*n* = 82, average adjusted *R^2^* 0.65 vs. 0.56, Mann–Whitney *U, p =* 0.046); however, only 29% of datasets were significantly improved by adding information on microbial community structure, by an average of 0.08 increase in adjusted *R^2^* within all models. Data on microbial community structure from ARISA, tRFLP, qPCR, and next generation technology did not differ in their explanatory power of process rates, but DGGE and PLFA had significantly lower explanatory power than other metrics (Supplementary Figure S2A). All microbial data types showed weak correlations to environmental variables but gene abundance data displayed a trend for higher correlation (*n* = 22, average adjusted *R^2^* 0.36) than community (*n* = 55, average adjusted *R^2^* 0.20) or functional diversity (*n* = 5, average adjusted *R^2^* 0.21) metrics (Supplementary Figure S2B).

**FIGURE 2:**
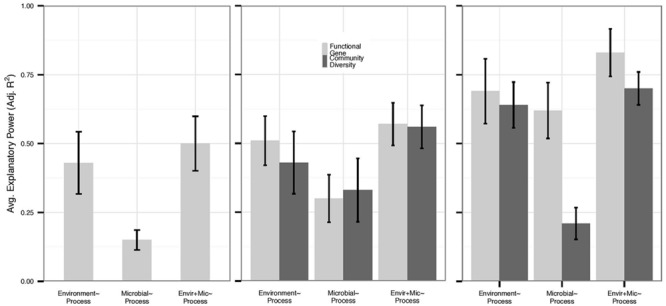
**Average explanatory power varied by process and microbial data type, and we present the explanatory power of functional genes (abundance and diversity) and community diversity for each N cycling process.** Environmental model bars slightly differ within each figure as they represent only the environmental models for each data type, and functional genes are not depicted for N mineralization due to low sample size (*n* = 2). Error bars represent standard errors. **(A)** N mineralization (*n* = 12) and **(B)** denitrification (*n* = 17) models had relatively low explanatory by all predictor sets. However, community diversity metrics (*n* = 4) provided more added value for denitrification rates than for other processes. **(C)** By contrast, rates of nitrification (*n* = 14) were well-explained by both environmental and functional gene predictor sets (*n* = 6) and were more likely to be improved by functional data.

We also examined each process individually for those functions for which we had the most data: nitrification (*n* = 14), denitrification (*n* = 17), N mineralization (*n* = 12), and respiration (*n* = 26). Overall, variation in N mineralization and denitrification rates was less well-explained than other processes by any predictor set (**Figure 2**). Only 17% of datasets examining N mineralization were improved by microbial community data – an average increase in adjusted *R^2^* of 0.008 within all N mineralization datasets – and the average explanatory power of microbial community structure alone on N mineralization rates was 0.21, compared to 0.31 in the full dataset. Within N mineralization studies that assayed microbial biomass, we found that microbial biomass correlated with environmental variables (*n* = 5, average adjusted *R^2^* 0.58) but not with process rates; none of the N mineralization models were improved with the addition of data on biomass. No denitrification studies examined microbial biomass or PLFAs. However, despite low replication, it is notable that in three of four studies, models of denitrification were improved with the addition of 16S rRNA gene diversity data (0.13 average increase in adjusted *R^2^*), while only three of eleven models were improved with data on functional gene abundance (0.04 average increase in adjusted *R^2^*).

Rates of nitrification were well-explained by all predictor sets (**Figure 2**). Nitrification models based on microbial data yielded the highest average adjusted *R^2^* of any process (0.38 across all data types and 0.50 if PLFA data are removed) with gene abundance (*n* = 4) data showing higher adjusted *R^2^* than community diversity data (*n* = 8; 0.61 vs. 0.21, Mann–Whitney *U, p* = 0.008). Moreover, 50% of environmental models of nitrification were improved with data on functional gene abundance or functional diversity (*n* = 6), while only 30% of models were improved with data on 16S rRNA gene diversity (*n* = 6), and PLFA data only improved 20% of models (*n* = 2). As with N mineralization, biomass was correlated with environmental variables (*n* = 3, average adjusted *R^2^* 0.62) and did not add explanatory power to nitrification rates.

**FIGURE 3:**
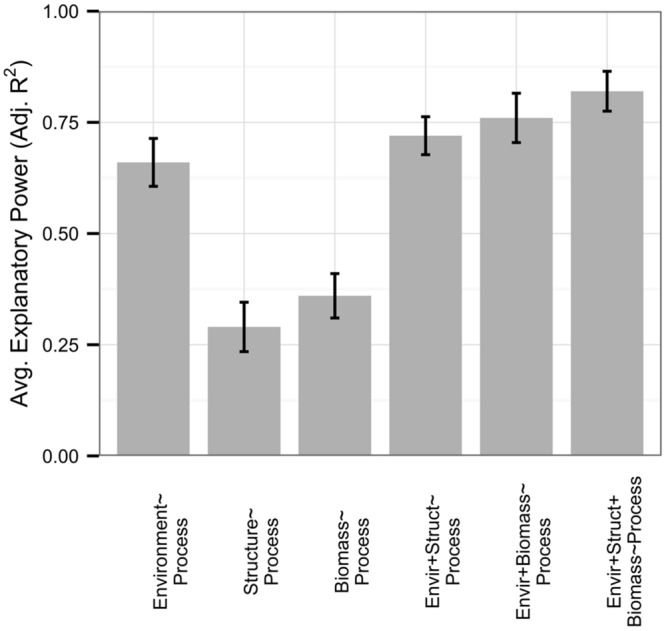
**Rates of respiration (*n* = 26) were well-explained by all predictor sets relative to the full dataset.** In this figure, we present microbial biomass in addition to microbial community structure, with standard errors represented as error bars. Microbial community structure and biomass independently had low explanatory power compared to environmental variables; however, they were largely uncorrelated with each other and provided greater model improvement when added to environmental variables in conjunction than when added independently.

Respiration rates were well-explained by the environment, with an average adjusted *R^2^* of 0.66 (**Figure 3**). Similar to the full dataset, microbial-only respiration models showed an average adjusted *R^2^* of 0.29 and only 23% of respiration models were improved with the addition of 16S rRNA gene diversity or PLFA data (no functional gene abundance datasets existed), by an average increase in 0.06 in adjusted *R^2^* across all respiration datasets. Interestingly, microbial biomass, when measured, improved 35% of models of respiration rates (*n* = 17, 0.09 average increase in adjusted *R^2^*), while 53% of models were improved with data on biomass and community structure in combination (0.15 average increase in adjusted *R^2^*). Microbial biomass was correlated to environmental variables with an average adjusted *R^2^* of 0.56 but not to microbial community structure (average adjusted *R^2^* 0.11).

**FIGURE 4:**
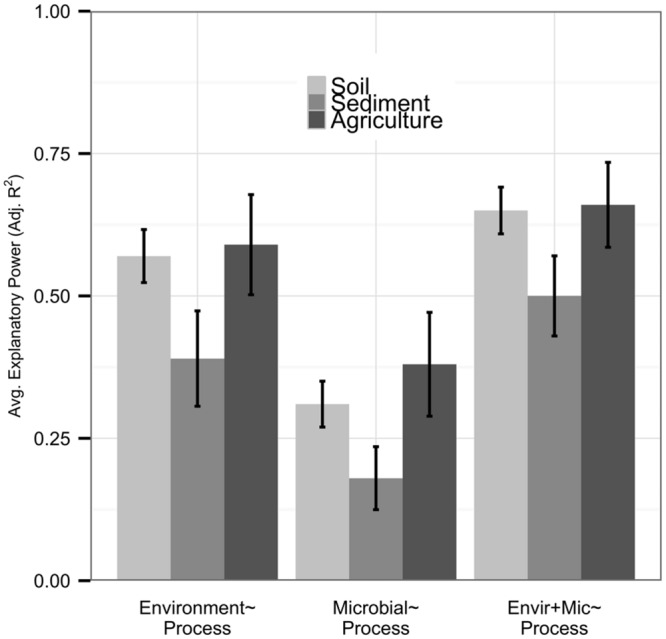
**We analyzed natural soil (*n* = 47), sediment (*n* = 12), and agricultural soil (*n* = 12) environments separately.** Bar height represents the average explanatory power of each predictor set, with error bars denoting standard errors. While predictor sets in natural soils nearly matched those within the full dataset, environmental and microbial predictor sets had lower explanatory power within sediments and higher explanatory power within agricultural soils. Microbial community diversity was less tightly coupled to the environment and provided more model improvement in sediments, with the opposite relationship in agricultural soils.

Although most of our datasets were from natural soil studies (*n* = 47), we had sufficient replication of sediment (*n* = 12) and agricultural soil (*n* = 12) datasets to examine these independently; other environments (*n* < 12) were excluded from this analysis. While statistically significant differences were not found, possibly due to the large variation in our datasets, we observed some notable trends across environments. Datasets from sediment samples were less well-explained by environmental or microbial predictor sets than other datasets (**Figure 4**). Relationships between community diversity and environmental conditions were also weak in sediment systems (*n* = 8, average adjusted *R^2^* 0.17), and community diversity metrics provided more added value (0.12 average increase in adjusted *R^2^*) in sediments than other systems. Agricultural systems had high explanatory power of process by both environmental (average adjusted *R^2^* 0.59) and microbial predictors (average adjusted *R^2^* 0.38) relative to other systems, and metrics of community diversity were more strongly correlated with the environment (*n* = 7, average adjusted *R^2^* 0.24) and had lower added value (average adjusted *R^2^* 0.06) than in other ecosystem types.

## Discussion

Despite the vast amount of variation in our datasets, our results indicate that data on microbial community structure may strengthen predictions of C and N cycling rates. Within our full dataset, environmental variables alone had greater explanatory power than either metrics of microbial community structure or microbial biomass but left 44% of variation in process rates unexplained on average (**Figure 1**). While we acknowledge that factors such as unmeasured variables, macroinvertebrates (Ferris et al., 2004), fungi (Talbot et al., 2008), plant communities (Hanson et al., 2000), and non-linearity could reduce the explanatory power of our models, a number of ecological dynamics can disassociate environmental conditions from microbial communities mediating C and N cycle processes and alter the relative value of environmental and microbial data for explaining rates of ecosystem processes (Knelman and Nemergut, 2014; Nemergut et al., 2014). When such factors strongly impact microbial communities, data on microbial community structure should enhance our predictions of ecosystem processes beyond those based solely on environmental data.

We observed stronger trends in the explanatory power of microbial predictors on ecosystem process rates when we reduced noise by examining subsets of data containing a single process or environment type. However, the lack of consistent trends within the full dataset is not surprising due to a variety of ecological and methodological factors. For instance, microorganisms can enter a state of lowered activity or dormancy in response to unfavorable environmental conditions (Jones and Lennon, 2010; Lennon and Jones, 2011), and research suggests that the percent of soil microbial communities catalyzing chemical reactions in soils at a given point in time can vary widely with resource history and disturbances (Blagodatskaya and Kuzyakov, 2013). These factors also induce spatially and temporally variable relationships between microbial communities and ecosystem processes, and the same microbial community may display different relationships to ecosystem function when sampled through time. Moreover, the extent to which functional traits are phylogenetically conserved varies with the trait of interest (Martiny et al., 2013), and thus, some processes may exhibit inherently stronger correlations with taxonomic metrics than others. Although, rapidly improving transcriptomic and proteomic sequencing technology may enhance predictions of ecosystem processes by identifying active segments of microbial communities (Abram, 2015), too few datasets presently exist to conduct a robust analysis with these data. Studies investigating the extent to which these dynamics are masked within observations of microbial community structure and function are imperative to deciphering and improving the current ability of microbial data to predict ecosystem process rates.

As well, discrepancies in methodology may also confound the relationship we observed in the full dataset between microbial communities and biogeochemical processes (Sinsabaugh et al., 2015). Microbial DNA is typically extracted from a fraction of gram of soil at a single time point and methods detect both dormant and active organisms, while corresponding process data are measured in several grams of soil over longer timeframes. Further, process rate measures are either collected as net measurements that aggregate multiple chemical reactions, potential rates that represent microbial response to substrate addition, or gross rates that trace individual chemical transformations (Bier et al., 2015). These approaches inevitably introduce data aggregation issues (Clark et al., 2011) and rely on assumptions that microbial and biogeochemical data are spatially and temporally representative. Recently, studies have attempted to reduce uncertainty associated with scalar differences by pairing spatially and temporally explicit field studies with ecological modeling approaches that interpolate data across scales (Lira-Noriega et al., 2013; Walker and Wardle, 2014). Leveraging these approaches in conjunction requires substantial resource investment but is increasingly feasible and presents a promising avenue for maximizing the value of microbial data.

Despite inherent limitations in microbial data, our data analysis suggests that microbial data types differ in their ability to explain processes that are phylogenetically broad vs. narrow and that are products of facultative vs. obligate metabolisms (**Figure 2**). Nitrification is the product of an obligate chemolithotrophic metabolism, and functional markers for this process are narrowly constrained within ammonia-oxidizing bacteria and archaea that catalyze the rate-limiting step in nitrification, putatively representing a likely dataset for detecting relationships between microbial data and process rates (Schimel, 1995; Schimel and Gulledge, 1998). While both functional gene abundance and community diversity metrics had similar average explanatory power within the full dataset (an effect that also did not vary among microbial technologies, Supplementary Figure S2A), models based on functional gene abundances or functional diversity explained more variation in process rates than community diversity within datasets measuring rates of nitrification (**Figure 2C**). Although these metrics were more strongly correlated with environmental factors than community diversity, they also provided greater explanatory power in combination with environmental variables than community diversity metrics, which include variation in many microbial guilds external to nitrification. Notably, models based on environmental variables alone also had higher explanatory power for nitrification than for denitrification or N mineralization, suggesting that the environment may also be more strongly coupled to nitrification rates than other N cycle processes. Taken together, these results suggest that despite a relatively tight connection between the environment, microbial community structure, and process data, the microbial guilds driving nitrification rates are partially decoupled at a functional level from environmental drivers of nitrification. Importantly, although nitrification was the only narrow process in our dataset with sufficient replication to examine independently, the high explanatory power of functional genes, both alone and in combination with environmental variables, for describing nitrification rates relative to other processes also conveys the potential for the inclusion of functional metrics in improving predictions of ecosystem processes that are mediated by phylogenetically narrow functional guilds.

By contrast, denitrification is a broad process, catalyzed by diverse facultative anaerobic microorganisms, and community diversity metrics provided more added value to denitrification rates than functional gene diversity or abundances (**Figure 2B**). While we acknowledge that genetic markers may not denote all functional genes involved in a process (Jones et al., 2014; Verbaendert et al., 2014), we observed more added value from community diversity metrics than functional genes despite several studies assaying up to three genes to explain denitrification rates, as opposed to one or two functional markers measured for nitrification. For broad processes such as denitrification, representations of niche complementarity or resource availability reflected in diversity metrics (Cadotte et al., 2011) rather than functional gene abundances that do not account for interactions between individuals (Barberán et al., 2012; Gagic et al., 2015) or functional redundancy (Allison and Martiny, 2008), may be important for explaining process rates. Thus, it is plausible that denitrifying communities are decoupled from environmental variables when measured at a broad taxonomic level and that such taxonomic metrics encompass more variation in within community interactions affecting denitrification rates than functional approaches. Although the sample size of datasets measuring denitrification was small, our analysis suggests that measurements of community diversity may be more likely than functional metrics to improve predictions of ecosystem processes that are catalyzed by facultative metabolisms and/or phylogenetically broad suites of organisms.

Similarly, the ability to respire carbon is widely distributed among microorganisms, and community diversity metrics (no datasets measured functional markers) described rates of respiration better than all other processes except denitrification (**Figure 3**). In fact, environmental and microbial variables, both alone and in combination, yielded higher average explanatory power of respiration rates than all other processes we examined independently with the exception of nitrification. Importantly, while recent work has suggested that microbial biomass (Schimel and Weintraub, 2003) or physiological properties such as drought tolerance (Manzoni et al., 2014) and growth efficiency (Wieder et al., 2013) can improve C cycling models, our results indicate that information on microbial community structure may further enhance our understanding of ecosystem C cycling. Microbial community structure and biomass appeared to explain differing portions of variation in process, as they exhibited extremely weak correlations with each other and, together, provided additive value for explaining rates of respiration. Thus, biomass and structure may jointly contribute to explaining rates of respiration as biomass may serve as a proxy for unmeasured environmental variables that regulate activity, such as soil structure (Gupta and Germida, 1988), while community structure may relate to the genetic capability of a community to respire carbon. Regardless, our results suggest that these data are at least partially independent and that measurements of microbial community structure could reduce uncertainty within Earth Systems models.

Lastly, the relationship between microbial community structure and ecosystem processes in our dataset varied by environment type (**Figure 4**). Sediment systems had weaker explanatory power of process rates by both environmental variables and microbial community structure than natural or agricultural soils. Sediment processes are influenced by both sediment and porewater chemistry (Middelburg and Levin, 2009), and these datasets may be more likely to have unmeasured variables or errors due to spatial discrepancies than other systems. In particular, dissolved oxygen concentration is a strong determinant of redox potential and ecosystem processes in sediments (Abell et al., 2011) but was only measured in three of 12 datasets. Despite the low explanatory power of sediment processes by all predictor sets, microbial community diversity added more value to explaining process rates and was more decoupled from environmental variables in sediments than in natural soil or agricultural systems, indicating that measurements of microbial community structure may improve predictions of sediment processes. Conversely, in agricultural soils, microbial community structure explained more variation in ecosystem processes but provided less improvement over environmental variables than other systems. Environmental variables were also better predictors of process in agricultural soils, and microbial community diversity was more tightly correlated with the environment than in sediments or natural soils. Agricultural systems homogenize soil structure and decrease soil organic matter (Dick, 1992; Chan and Heenan, 1996), reducing the number and complexity of microbial niches in agricultural soils. Thus, relatively coarse-scale measures of environmental conditions may more directly correlate with both community structure and ecosystem process rates than in more variable systems. Overall, these results indicate that ecosystem-specific dynamics may be crucial to understanding the value of microbial community structure for explaining ecosystem processes and emphasize the importance of future investigations into understudied biomes in enhancing our understanding of global relationships between microbial communities and ecosystem processes.

Here, we present the first extensive investigation into the relationship between environmental conditions, microbial community structure, and ecosystem function by re-analyzing 82 datasets collected from an international team of collaborators, and we demonstrate that nuanced relationships between the environment and microbial communities can influence the importance of microbial community structure for explaining ecosystem-level processes. Our analysis provides an empirical basis for future hypothesis-testing on the roles of assembly, dormancy, redundancy and phenotypic plasticity in microbial community structure and function, and despite complexity in our datasets, the trends we observed represent an encouraging step for linking microbial community data to ecosystem function. As a whole, our results indicate that a greater understanding of microbial community structure informed by ecological principles may further our ability to accurately predict rates of ecosystem processes beyond environmental variables and bulk physiological characterizations of microbial communities.

## Author Contributions

All authors collected and contributed datasets for analysis as well as participated in the conceptual drafting and revision of this manuscript. EG conducted all data analysis and was the primary author in writing and revising the manuscript. DN and JK contributed significantly in manuscript development and revision, and DN provided financial support for this work.

## Conflict of Interest Statement

The authors declare that the research was conducted in the absence of any commercial or financial relationships that could be construed as a potential conflict of interest.
