# Supplementary material for: Microbes as Engines of Ecosystem Function: When Does Community Structure Enhance Predictions of Ecosystem Processes?
Source: Front Microbiol. 2016 Feb 24;7:214. doi: 10.3389/fmicb.2016.00214 (PMC4764795; doi:10.3389/fmicb.2016.00214)
Supplement: Supplementary file 1 [file Data_Sheet_1.DOCX]

**SUPPLEMENTAL METHODS**

**Data collection**

Our search terms included a variety of environmental, microbial, and process parameters encompassing a broad swath of studies constrained by date from 2005 to late 2013. We searched the Web of Science database by topic for the following terms: ‘community assembly + process + microb*’, ‘community assembly + carbon cycle + microb*’, ‘community assembly + carbon rate + microb*’, ‘community assembly + nitrogen cycle + microb*’, ‘community assembly + nitrogen rate + microb*’, ‘community assembly + biogeochem* + microb*’, ‘community assembly + biogeochem* + microb*’, ‘microb* + biogeochem* + process’, ‘16S + biogeochem*’, ‘16S + carbon cycle’, ‘16S + carbon rate’, ‘16S + nitrogen cycle’, ‘16S + nitrogen rate’, ‘qPCR + biogeochem*’, ‘qPCR + carbon cycle’, ‘qPCR + carbon rate’, ‘qPCR + nitrogen cycle’, ‘qPCR + nitrogen rate.’ While our results yielded thousands of potential studies, only 117 contained all three sets of data, and we contacted each author to acquire the original data. Datasets matching our criteria were also harvested from the Earth Microbiome Project Database. While we were able to obtain 106 datasets, only 82 contained sufficient replication (n ≥12) of appropriate variables for our analysis.

We examined datasets mostly from marine systems, tropical forests, arctic permafrost, freshwater wetlands, and agricultural systems, though the majority of our datasets were from temperate soil samples. Most datasets contained data on nitrification, denitrification, nitrogen (N) mineralization, or carbon mineralization (broadly referred to here as ‘respiration’), but we also included datasets with methanogenesis, methane oxidation, primary productivity, dissimilatory nitrate reduction to ammonium, or nitrogen fixation as a process. We accepted PLFA and DNA fingerprinting (DGGE, tRFLP, ARISA) of 16S rRNA or functional genes, targeted functional gene (qPCR), or next generation sequencing (454 pyrosequencing) techniques.

**Statistical Modeling**

*Data types*

We included standing pools of nutrients and carbon, pH, temperature, moisture, and oxygen, as well as categorical variables such as site or vegetation type, into our models as environmental variables. Multivariate environmental predictors such as soil texture and soil carbon chemistry were collapsed into Euclidean distance matrices, and the first principle coordinate was included as an environmental predictor following the procedure detailed below for microbial community structure data.

Data from PLFA, DNA fingerprinting techniques, and next generation sequencing were distilled into Bray-Curtis distance matrices using the ‘vegan’ package (Oksanen et al., 2005) in *R* software (R Core Team, 2014) or ‘beta_diversity.py’ command in QIIME (Caporaso et al., 2010). Then, the first three principle coordinates of each distance matrix (explaining a median of 70% variation, standard deviation 17%) were extracted using the ‘labdsv’ package (Roberts, 2007) in *R* and included as predictors in the model as measures of community dissimilarity. For functional gene diversities, if multiple functional genes were measured, only the first two principle coordinates (explaining a median of 70% variation per gene, standard deviation 17%) from each gene were included due to processing power limitations. We included functional gene abundances as relative abundances, and there was no difference in the explanatory power of datasets with total versus relative functional gene abundances. We also examined nine studies in which it was possible to calculate both total and relative gene abundances and found no differences in explanatory power of each type of data (data not shown).

*Multimodel Inference*

For each dataset, the distribution of each variable was assessed using a histogram and a Shapiro-Wilk normality test (Shapiro and Wilk, 1965), and variables were natural log transformed when *p* < 0.05. We constructed a global linear regression model for each predictor set with the measured process rate as the dependent variable and all measured predictors as independent variables. Interaction terms between independent variables were not included both to maintain consistency in variables across studies and because we did not possess system-specific ecological justification for their inclusion. We also decided against altering predictor sets to address colinearity between independent variables, as our objective was to maximize the explanatory power of each predictor set rather than to determine the explanatory power of individual variables, and our statistical approach partially compensated for colinearity by penalizing models with more independent variables.

Using the ‘dredge’ command in the ‘MuMIn’ package to evaluate the explanatory power of all possible combinations of predictors within each global model, we selected a set of best fit models for each process containing all models with a delta AICc value no more than four greater than the model with the lowest AICc value. Predictor set lengths were limited to three less than the number of data points to minimize model overfitting. A weighted average of these best fit models, using Akaike’s weights to estimate the likelihood of each model, was chosen as our final model for each predictor class. The use of Akaike’s weights assigns lower importance to models with greater delta AICc values, lessening their impact on the parameters in the averaged final model.

To validate our approach, we constructed best fit models using stepwise forwards regressions with data from three study systems for which we possessed a detailed ecological understanding. These datasets examined four different biogeochemical processes using either qPCR of functional genes or next generation sequencing to measure microbial community structure, and our models allowed for independent variables to interact when ecologically relevant to represent the best possible expert-built models. All models closely matched those generated by multimodel inference, with average differences in adjusted *R^2^* values of 0.03 for models based on environmental variables, 0.06 for models based on microbial variables (strongly influenced by one model in which both methods yielded low (<0.25) adjusted *R^2^* values), and 0.01 for models based on both environmental and microbial variables.

*Redundancy Analysis*

We examined correlations between predictor sets (environmental vs. microbial community structure, environmental vs. microbial biomass, microbial biomass vs. community structure) using redundancy analysis (RDA). Microbial community composition data but not functional gene abundances were Hellinger transformed (Legendre and Gallagher, 2001) to reduce asymmetry of the data, and both were treated as the dependent variable in all cases. Microbial biomass was untransformed and treated as the dependent variable in analyses with environmental variables and as the independent variable in models with microbial community structure. All environmental predictors in the final environmental models were used as independent variables for this analysis.

**References**

Caporaso, J.G., Kuczynski, J., Stombaugh, J., Bittinger, K., Bushman, F.D., Costello, E.K., Fierer, N., Pena, A.G., Goodrich, J.K., and Gordon, J.I. (2010). QIIME allows analysis of high-throughput community sequencing data. *Nature methods* 7**,** 335-336.

Legendre, P., and Gallagher, E.D. (2001). Ecologically meaningful transformations for ordination of species data. *Oecologia* 129**,** 271-280.

Oksanen, J., Kindt, R., and O’Hara, B. (2005). Vegan: R functions for vegetation ecologists. *URL:* [*http://cc*](http://cc)*.oulu.fi/jarioksa/softhelp/vegan.html*.

R Core Team (2014). "R: A Language and Environment for Statistical Computing". (Vienna, Austria: R Foundation for Statistical Computing).

Roberts, D. (2007). labdsv: Ordination and multivariate analysis for ecology. *R package*.

Shapiro, S.S., and Wilk, M.B. (1965). An analysis of variance test for normality (complete samples). *Biometrika***,** 591-611.

**SUPPLEMENTAL FIGURES AND TABLE**

**Supplementary Figure 1** We employed a four-step method for each dataset to analyze and compare the explanatory power of each predictor set. (1) To pre-process our data, multivariate environmental and microbial data were collapsed into Euclidean and Bray-Curtis distance matrices, respectively, and principal coordinate analysis (PCoA) was utilized to vectorize the data. The Shannon diversity index was also calculated from multivariate microbial data, and all variables were checked for normality and natural log transformed as necessary. (2) We used a multimodel inference approach to generate an averaged linear regression model for each predictor set, and (3) then we compared models within each study across predictor sets with a statistical significance criterion of a delta AICc of four and an ecological significance criterion of 10% improved in adj. *R^2^*. (4) Finally, we synthesized and compared results across relevant subsets of datasets using our significance criteria, as well as the adj. *R^2^* value for each dataset.

**Supplementary Figure 2** When we analyzed data by microbial data type, (a) PLFA (*n* = 20) and DGGE (*n* = 7) had significantly lower explanatory power on process rates than other DNA fingerprinting (ARISA *n* = 4, tRFLP functional gene *n* = 11, tRFLP 16S *n* = 11), qPCR (n = 22), or next generation sequencing techniques (*n* = 11). (b) Functional gene abundances (*n* = 22) displayed a trend for higher correlations with environmental variables than community (*n* = 55) or functional (*n* = 5) diversity. Bar height represents the average adj. *R^2^* value, and error bars denote standard errors.

**Supplementary Table 1. Summary of Re-analyzed Datasets**

 * = Statistical Improvement

+ = Ecological Improvement

 * = Statistical Improvement

+ = Ecological Improvement

**References for Supplementary Table 1**

Abell, G.C., Stralis‐Pavese, N., Sessitsch, A., and Bodrossy, L. (2009). Grazing affects methanotroph activity and diversity in an alpine meadow soil. *Environmental microbiology reports* 1**,** 457-465.

Andert, J., Wessén, E., Börjesson, G., and Hallin, S. (2011). Temporal changes in abundance and composition of ammonia-oxidizing bacterial and archaeal communities in a drained peat soil in relation to N_2_O emissions. *Journal of Soils and Sediments* 11**,** 1399-1407.

Angel, R., Claus, P., and Conrad, R. (2012). Methanogenic archaea are globally ubiquitous in aerated soils and become active under wet anoxic conditions. *The ISME journal* 6**,** 847-862.

Bañeras, L., Ruiz-Rueda, O., López-Flores, R., Quintana, X., and Hallin, S. (2012). The role of plant type and salinity in the selection for the denitrifying community structure in the rhizosphere of wetland vegetation. *International Microbiology* 15**,** 89-99.

Banerjee, S., and Siciliano, S.D. (2012). Factors driving potential ammonia oxidation in Canadian arctic ecosystems: does spatial scale matter? *Applied and environmental microbiology* 78**,** 346-353.

Biasi, C., Meyer, H., Rusalimova, O., Hämmerle, R., Kaiser, C., Baranyi, C., Daims, H., Lashchinsky, N., Barsukov, P., and Richter, A. (2008). Initial effects of experimental warming on carbon exchange rates, plant growth and microbial dynamics of a lichen-rich dwarf shrub tundra in Siberia. *Plant and Soil* 307**,** 191-205.

Cao, Y., Green, P.G., and Holden, P.A. (2008). Microbial community composition and denitrifying enzyme activities in salt marsh sediments. *Applied and environmental microbiology* 74**,** 7585-7595.

Chatterjee, A., Ingram, L., Vance, G., and Stahl, P. (2009). Soil processes and microbial community structures in 45-and 135-year-old lodgepole pine stands. *Canadian journal of forest research* 39**,** 2263-2271.

Cheneby, D., Brauman, A., Rabary, B., and Philippot, L. (2009). Differential responses of nitrate reducer community size, structure, and activity to tillage systems. *Applied and environmental microbiology* 75**,** 3180-3186.

Foulquier, A., Volat, B., Neyra, M., Bornette, G., and Montuelle, B. (2013). Long-term impact of hydrological regime on structure and functions of microbial communities in riverine wetland sediments. *FEMS microbiology ecology* 85**,** 211-226.

Garcia-Pausas, J., and Paterson, E. (2011). Microbial community abundance and structure are determinants of soil organic matter mineralisation in the presence of labile carbon. *Soil Biology and Biochemistry* 43**,** 1705-1713.

Goberna, M., Garcia, C., Insam, H., Hernández, M., and Verdú, M. (2012). Burning fire-prone Mediterranean shrublands: immediate changes in soil microbial community structure and ecosystem functions. *Microbial ecology* 64**,** 242-255.

Godin, A., McLaughlin, J.W., Webster, K.L., Packalen, M., and Basiliko, N. (2012). Methane and methanogen community dynamics across a boreal peatland nutrient gradient. *Soil Biology and Biochemistry* 48**,** 96-105.

Hallin, S., Jones, C.M., Schloter, M., and Philippot, L. (2009). Relationship between N-cycling communities and ecosystem functioning in a 50-year-old fertilization experiment. *The ISME journal* 3**,** 597-605.

Hamer, U., and Makeschin, F. (2009). Rhizosphere soil microbial community structure and microbial activity in set-aside and intensively managed arable land. *Plant and soil* 316**,** 57-69.

Ingram, L., Stahl, P., Schuman, G., Buyer, J., Vance, G., Ganjegunte, G., Welker, J., and Derner, J. (2008). Grazing impacts on soil carbon and microbial communities in a mixed-grass ecosystem. *Soil Science Society of America Journal* 72**,** 939-948.

Isobe, K., Koba, K., Suwa, Y., Ikutani, J., Fang, Y., Yoh, M., Mo, J., Otsuka, S., and Senoo, K. (2012). High abundance of ammonia-oxidizing archaea in acidified subtropical forest soils in southern China after long-term N deposition. *FEMS microbiology ecology* 80**,** 193-203.

Kaisermann, A., Roguet, A., Nunan, N., Maron, P.-A., Ostle, N., and Lata, J.-C. (2013). Agricultural management affects the response of soil bacterial community structure and respiration to water-stress. *Soil Biology and Biochemistry* 66**,** 69-77.

Knelman, J.E., Legg, T.M., O’Neill, S.P., Washenberger, C.L., González, A., Cleveland, C.C., and Nemergut, D.R. (2012). Bacterial community structure and function change in association with colonizer plants during early primary succession in a glacier forefield. *Soil Biology and Biochemistry* 46**,** 172-180.

Koranda, M., Schnecker, J., Kaiser, C., Fuchslueger, L., Kitzler, B., Stange, C.F., Sessitsch, A., Zechmeister-Boltenstern, S., and Richter, A. (2011). Microbial processes and community composition in the rhizosphere of European beech–the influence of plant C exudates. *Soil Biology and Biochemistry* 43**,** 551-558.

Lamb, E.G., Han, S., Lanoil, B.D., Henry, G.H., Brummell, M.E., Banerjee, S., and Siciliano, S.D. (2011). A High Arctic soil ecosystem resists long‐term environmental manipulations. *Global Change Biology* 17**,** 3187-3194.

Lauber, C.L., Strickland, M.S., Bradford, M.A., and Fierer, N. (2008). The influence of soil properties on the structure of bacterial and fungal communities across land-use types. *Soil Biology and Biochemistry* 40**,** 2407-2415.

Leff, J.W., Nemergut, D.R., Grandy, A.S., O’Neill, S.P., Wickings, K., Townsend, A.R., and Cleveland, C.C. (2012). The effects of soil bacterial community structure on decomposition in a tropical rain forest. *Ecosystems* 15**,** 284-298.

Lindström, E.S., Feng, X.M., Granéli, W., and Kritzberg, E.S. (2010). The interplay between bacterial community composition and the environment determining function of inland water bacteria. *Limnology and Oceanography* 55**,** 2052-2060.

Lopes, A.R., Faria, C., Prieto-Fernández, Á., Trasar-Cepeda, C., Manaia, C.M., and Nunes, O.C. (2011). Comparative study of the microbial diversity of bulk paddy soil of two rice fields subjected to organic and conventional farming. *Soil Biology and Biochemistry* 43**,** 115-125.

Miller, M., Zebarth, B., Dandie, C., Burton, D., Goyer, C., and Trevors, J. (2008). Crop residue influence on denitrification, N_2_O emissions and denitrifier community abundance in soil. *Soil Biology and Biochemistry* 40**,** 2553-2562.

Nogaro, G., Datry, T., Mermillod‐Blondin, F., Foulquier, A., and Montuelle, B. (2013). Influence of hyporheic zone characteristics on the structure and activity of microbial assemblages. *Freshwater Biology* 58**,** 2567-2583.

Papaspyrou, S., Smith, C.J., Dong, L.F., Whitby, C., Dumbrell, A.J., and Nedwell, D.B. (2014). Nitrate reduction functional genes and nitrate reduction potentials persist in deeper estuarine sediments. Why. *PloS one* 9.

Petersen, D.G., Blazewicz, S.J., Firestone, M., Herman, D.J., Turetsky, M., and Waldrop, M. (2012). Abundance of microbial genes associated with nitrogen cycling as indices of biogeochemical process rates across a vegetation gradient in Alaska. *Environmental microbiology* 14**,** 993-1008.

Philippot, L., Čuhel, J., Saby, N., Chèneby, D., Chroňáková, A., Bru, D., Arrouays, D., Martin‐Laurent, F., and Šimek, M. (2009). Mapping field‐scale spatial patterns of size and activity of the denitrifier community. *Environmental microbiology* 11**,** 1518-1526.

Potthast, K., Hamer, U., and Makeschin, F. (2012a). In an Ecuadorian pasture soil the growth of Setaria sphacelata, but not of soil microorganisms, is co-limited by N and P. *Applied Soil Ecology* 62**,** 103-114.

Potthast, K., Hamer, U., and Makeschin, F. (2012b). Land-use change in a tropical mountain rainforest region of southern Ecuador affects soil microorganisms and nutrient cycling. *Biogeochemistry* 111**,** 151-167.

Schindlbacher, A., Rodler, A., Kuffner, M., Kitzler, B., Sessitsch, A., and Zechmeister-Boltenstern, S. (2011). Experimental warming effects on the microbial community of a temperate mountain forest soil. *Soil Biology and Biochemistry* 43**,** 1417-1425.

Smithwick, E.A., Naithani, K.J., Balser, T.C., Romme, W.H., and Turner, M.G. (2012). Post-Fire spatial patterns of soil nitrogen mineralization and microbial abundance. *PloS one* 7**,** e50597.

Strickland, M.S., Callaham, M.A., Davies, C.A., Lauber, C.L., Ramirez, K., Richter, D.D., Fierer, N., and Bradford, M.A. (2010). Rates of in situ carbon mineralization in relation to land-use, microbial community and edaphic characteristics. *Soil Biology and Biochemistry* 42**,** 260-269.

Thomson, B.C., Ostle, N., McNamara, N., Bailey, M.J., Whiteley, A.S., and Griffiths, R.I. (2010). Vegetation affects the relative abundances of dominant soil bacterial taxa and soil respiration rates in an upland grassland soil. *Microbial ecology* 59**,** 335-343.

Wessén, E., Nyberg, K., Jansson, J.K., and Hallin, S. (2010). Responses of bacterial and archaeal ammonia oxidizers to soil organic and fertilizer amendments under long-term management. *Applied Soil Ecology* 45**,** 193-200.

Wessén, E., Söderström, M., Stenberg, M., Bru, D., Hellman, M., Welsh, A., Thomsen, F., Klemedtson, L., Philippot, L., and Hallin, S. (2011). Spatial distribution of ammonia-oxidizing bacteria and archaea across a 44-hectare farm related to ecosystem functioning. *The ISME journal* 5**,** 1213-1225.

Widmer, F., Rasche, F., Hartmann, M., and Fliessbach, A. (2006). Community structures and substrate utilization of bacteria in soils from organic and conventional farming systems of the DOK long-term field experiment. *Applied Soil Ecology* 33**,** 294-307.

Wieder, W.R., Cleveland, C.C., Taylor, P.G., Nemergut, D.R., Hinckley, E.-L., Philippot, L., Bru, D., Weintraub, S.R., Martin, M., and Townsend, A.R. (2013). Experimental removal and addition of leaf litter inputs reduces nitrate production and loss in a lowland tropical forest. *Biogeochemistry* 113**,** 629-642.

**Funding Sources:**

ESF EuroDiversity programme METHECO; FWF, Austria; Swedish Research Council Formas; Giessen FACE Experiment; Institute for Water Research of Ben-Gurion University of the Negev, Israel; Arava Institute for Environmental Studies, Israel; The Institute for Soil Science and Forest Nutrition (IBW); Max Planck Society; Spanish Ministry of Science and Innovation; the Doñana Biological Station; Climate Change Impacts on Canadian Arctic Tundra (CiCAT); International Polar Year (IPY); Austrian Academy of Science; Austrian Federal Ministry of Education, Science and Culture (PayerWeyprecht Society); YamburgGazDobycha (a subsidiary company of Gazprom, Russia); U.S. Environmental Protection Agency's STAR Estuarine and Great Lakes (EaGLe); National Science Foundation; ACI-FNS ECCO program MUTEN from the French Ministry of Research; French National Research Agency; Agència de Gestió d’Ajuts Universitaris i de Recerca” (AGAUR) of the Catalan Government; the James Hutton Institute; EU Marie Curie Programme; VAMPIRO; Linktree; Natural Sciences and Engineering Research Council of Canada Discovery Grant; the Ontario Forest Research Institute, Ontario Ministry of Natural Resources (OMNR); Helmholtz Centre for Environmental Research-UFZ (Halle, Germany); National Research Initiative grant from the Cooperative State Research, Education, and Extension Service, U.S. Department of Agriculture; Mitsui & Co., Ltd. Environment Fund; Scientific Research from the Ministry of Education, Culture, Sports, Science, and Technology, Japan; the National Natural Science Foundation of China; the Grant for Projects for the Protection, Preservation, & Restoration of Cultural Properties in Japan by the Sumitomo Foundation; NEXT Program; Région Ile de France via the R2DS Funding Program; the “Centre National pour la Recherche Scientifique via the INSU Funding Program”; National Science Foundation (NSF); Austrian Science Fund; CiCAT; ArcticNet; Andrew W. Mellon Foundation; Crafoord Foundation; Fundação para a Ciência e a Tecnologia; the Xunta de Galicia; GAPS Program of Agriculture and Agri-Food Canada (AAFC); the Carlsberg foundation; the Bonanza Creek Long-Term Ecological Research program; the Danish National Research Foundation; Ministère des Affaires Etrangères et Européennes in France; and by Ministry of Education, Youth and Sports in the Czech Republic; the Grant Agency of the AS CR; the Office of Science (BER), U.S. Department of Energy; Lawrence Berkeley National Laboratory; the Swedish Farmers’ Foundation for Agricultural Research; Swiss Federal Office for Agriculture; the Swiss National Science Foundation
